# Scoping review: Facilitators, barriers, and cultural adaptations in the caregiver skills training program for children with developmental concerns

**DOI:** 10.1177/13623613251406399

**Published:** 2025-12-27

**Authors:** Cecilia Montiel-Nava, Maria C Montenegro, Ana C Ramirez, Victoria Villarreal, Lucia Murillo Chacko, Pamela Dixon, Sarah Dababnah

**Affiliations:** 1 The University of Texas Rio Grande Valley, USA; 2 Autism Speaks, USA; 3 University of Maryland, USA

**Keywords:** autism, cultural adaptation, developmental disabilities, parent-mediated interventions, task-shifting

## Abstract

Autism interventions are predominantly developed in high-income countries, limiting access for families in low- and middle-income countries due to systemic, cultural, and logistical barriers. The Caregiver Skills Training program aims to address this disparity by equipping caregivers with practical skills. This scoping review examines the cultural adaptations, facilitators, and barriers to the implementation of Caregiver Skills Training, focusing on its accessibility, feasibility, and acceptability. A comprehensive search of ERIC, PsycINFO, PubMed, and Web of Science identified eligible studies that reported cultural or linguistic adaptations of Caregiver Skills Training. Forward searches and manual reference checks supplemented the review. Data were extracted using the Cultural Adaptation Checklist framework and analyzed for patterns in adaptation, training, barriers, and facilitators. Seventeen studies across Asia, Africa, Europe, and North America highlighted diverse adaptations in language, content, and delivery methods. Facilitators included community partnerships and task-shifting with non-specialists, while barriers involved logistical challenges, stigma, and resource constraints. Caregiver Skills Training’s flexible, culturally responsive framework makes it a viable model for scaling autism interventions globally. Tailored adaptations and strong support systems for facilitators are essential to overcoming systemic challenges and ensuring equitable access in low- and middle-income countries.

**Lay abstract:**

**How the Caregiver Skills Training Program Helps Families Worldwide**

The Caregiver Skills Training program was designed to help families of children with autism and other developmental challenges in low-resource settings. Caregiver Skills Training empowers parents and caregivers by teaching them practical strategies to improve their child’s communication, social interaction, and daily living skills. This program is unique because it does not require a formal diagnosis and is designed to be delivered by trained non-specialists, such as community health workers. A review of 17 studies from different countries examined how the Caregiver Skills Training program was adapted to fit the cultural and practical needs of families in each region. For example, materials were translated, simplified, and paired with visual aids to help parents with lower literacy levels. Non-specialist facilitators helped make the program more accessible, and online or hybrid delivery methods increased participation during the COVID-19 pandemic. However, challenges remain. Families often face barriers like limited transportation, stigma, and lack of Internet access, which can prevent them from fully participating in the program. Facilitators also need more training and support to maintain program quality. Despite these obstacles, Caregiver Skills Training shows promise as a global solution to bridge the gap in autism care, especially in underserved communities. This review highlights the importance of adapting programs like Caregiver Skills Training to meet the unique needs of families worldwide, ensuring that every child has the opportunity to thrive, regardless of where they live.

Autism Spectrum Disorder (ASD; hereafter, autism) is a developmental condition that encompasses diverse ways of thinking, learning, and interacting with the world (American Psychiatric Association, 2022). Research demonstrates that early intervention, particularly in the first few years of life, significantly improves social skills, communication, and adaptive behavior (Estes et al., 2015; Zwaigenbaum et al., 2015), with access to behavioral, speech, and language therapy enhancing developmental outcomes and quality of life (Dawson et al., 2010; Landa, 2018). Timely, appropriate care is thus essential for empowering autistic children to thrive and participate in their communities.

Despite consistent autism prevalence across races/ethnicities (Maenner et al., 2023; Nowell et al., 2015; Zeidan et al., 2022), disparities in diagnosis and service access persist, particularly in low- and middle-income countries (LMICs) (Montiel-Nava et al., 2020). Limited healthcare infrastructure, few professionals, and socioeconomic barriers hinder interventions (Aylward et al., 2021; Divan et al., 2021; Durkin et al., 2015; Malik-Soni et al., 2022). Poverty and transportation issues exacerbate inequities (Bishop-Fitzpatrick & Kind, 2017). Autism research, concentrated in high-income countries (HICs), limits cultural understanding (de Leeuw et al., 2020; Divan et al., 2024; Montiel-Nava et al., 2023). In LMICs, shortages of specialists and fragmented services, particularly in rural areas, lead to long waitlists and delayed interventions (Daniels & Mandell, 2014; Montiel-Nava et al., 2020; Vohra et al., 2014; Zuckerman et al., 2015).

Parent-mediated interventions (PMIs) address disparities in autism care by teaching parents strategies to support communication, social engagement, and daily living skills in natural settings (Oono et al., 2013). Non-specialists, such as community health workers, can effectively deliver PMIs (Althoff et al., 2019; Cherewick et al., 2023; McConachie & Diggle, 2007; Naithani et al., 2022; Reichow et al., 2013), with cultural adaptations via Community-Based Participatory Research enhancing feasibility(Fletcher-Watson et al., 2019). However, PMI research often focuses on White, affluent populations (de Leeuw et al., 2020; Divan et al., 2021; Pervin et al., 2022), making specialist-led interventions unfeasible in low-resource settings due to cost and access barriers (Montiel-Nava et al., 2020; Naithani et al., 2022). The Caregiver Skills Training (CST) program uses task-shifting to train non-specialists, like community health workers and family volunteers, addressing the shortage of autism intervention professionals and enhancing scalability in LMICs with a transdiagnostic focus and common elements approach (Salomone et al., 2019; Sengupta et al., 2023; Tekola et al., 2020).

This scoping review aims to investigate how cultural adaptations and task-shifting strategies impact the implementation, accessibility, and effectiveness of the CST program for autistic children and their families in diverse cultural and resource-constrained settings, while identifying common elements and site-specific factors influencing outcomes. Prior syntheses of PMIs and CST provide context but highlight gaps. Oono et al. (2013) conducted a Cochrane review of PMIs, focusing on the efficacy of early intervention in high-income settings, with limited attention to cultural adaptations. Reichow et al. (2024) performed a systematic review and meta-analysis, showing improvements in child development and parenting skills, primarily in high-income contexts with less focus on cultural adaptations in LMICs. Naithani et al. (2022) explored non-specialist delivery in LMICs but lacked CST-specific global insights. Kamaralzaman et al. (2022) explored the feasibility of implementing the CST adaptation in Malaysia by reviewing feasibility studies from India, Ethiopia, and Italy; however, their scope was confined to a single country’s implementation. Our review addresses these gaps by synthesizing CST-specific cultural adaptations and task-shifting approaches across different studies from diverse regions, offering novel insights into the global scalability and equity of CST.

Cultural adaptation—defined as language translation, content modification to reflect local values and practices, delivery method adjustments (e.g. oral vs written formats, in-person vs online), and integration of community-specific beliefs and caregiving norms (Castro et al., 2023; Domenech Rodríguez et al., 2011)—is critical for PMIs like CST to align with diverse populations while maintaining evidence-based integrity (Albin et al., 2022; Soto & Vega, 2024). Such adaptations reduce health disparities and improve outcomes (Cycyk et al., 2021). However, limited guidance exists for adapting PMIs in low-resource settings. Since 2016, CST has been implemented in over 40 countries with varied adaptations, supporting the need for this review.

## Method

We conducted a scoping review following PRISMA-ScR guidelines (“PRISMA,” 2015; Tricco et al., 2018), including English or Spanish articles on CST adaptation/implementation. Studies not detailing cultural/linguistic adaptations were excluded.

### Search strategy

**Figure 1.**
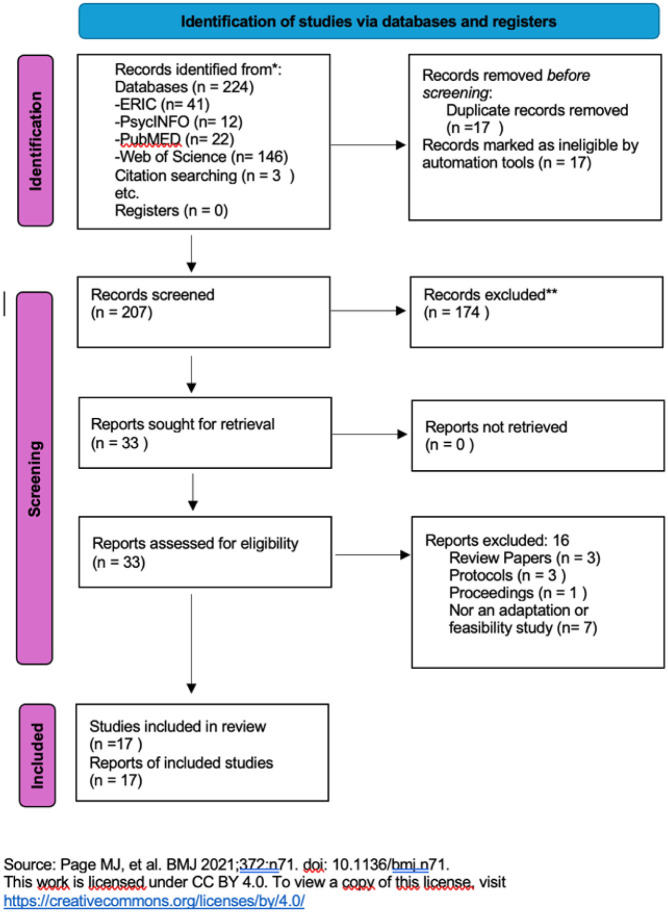
PRISMA flow chart.

We searched ERIC, PsycINFO, PubMed, and Web of Science in August 2025, using a team-refined strategy with Boolean keywords: (World Health Organization Caregiver Skills Training) AND (Developmental Delays OR autism-related terms) AND (adaptation OR feasibility OR acceptability). These terms captured barriers/facilitators from full texts. Since this program was first released for field testing in 2016, and the first article was published in 2018, we searched articles published from 2018 to August 2025. Records were uploaded to EndNote for duplicate checking (Figure 1).

### Data extraction and quality assessment

We extracted studies meeting eligibility criteria using a chart that included all the Cultural Adaptation Checklist (CAC) domains (https://osf.io/n3gf8/?view_only=57ea77bca5b84f7b8b63525efd8e26f2). Two reviewers used this chart to code the data independently, reviewed the results using an iterative process, and updated the table as they reached agreements. Discrepancies were resolved through discussion and consensus, with input from a third team member when needed. This iterative process ensured consistency and reliability in coding decisions. We used the Ecological Validity Framework (EVF; Bernal et al., 1995) and the CAC (Lee et al., 2023) to determine CST adaptation features. The CAC is based on the EVF and outlines seven key dimensions for effective cultural adaptation of evidence-based interventions for children with developmental disabilities, including autism(Lee et al., 2023), making it suitable to assess the cultural adaptation process of CST: (1) *Language*: Translating materials into multiple languages when necessary and using terminology familiar to the target population. (2) *Persons*: Establishing strong relationships with community organizations and stakeholders and collaborating with them throughout all phases of the intervention. (3) *Content*: Ensuring the information provided has practical relevance and cultural significance for the target population. (4) *Goals*: Ensuring that the intervention’s goals align with the priorities and needs of the target population. (5) *Methods*: Implementing the intervention in a way that integrates the specific needs and preferences of the target population. (6) *Context*: Considering the influence of geographical, economic, and political factors that shape the lives of the target population. (7) *Process*: Actively soliciting feedback from community stakeholders and integrating it into the adaptation process for continuous improvement.

We utilized Lee et al. (2023) criteria for each domain, with the exception of “Goals.” All studies focused on the same intervention and shared goals of fostering joint engagement, communication, adaptive behavior responses, and caregiver well-being. In addition, we coded all studies in terms of (a) study characteristics (author(s), year of publication, country, language, delivery method, setting, study design, sample size, age of children and diagnosis, outcome measures); (b) training information (interventionist supervision and fidelity); (c) barriers (challenges hindering feasibility and acceptability); (d) facilitators (factors increasing acceptability and feasibility); and (e) suggestions for improving the program implementation (see supplemental materials). We primarily applied deductive codes based on the CAC and EVF; however, inductive codes also emerged during iterative review (e.g. “toy kits,” “privacy concerns,” “role-play discomfort”), which were integrated into the final synthesis. We identified themes through repeated review and consensus discussions among coders. We used a matrix-based approach to compare patterns across studies and domains, which informed the synthesis presented in the results and Table 1. We charted the data using Microsoft Excel.

**Table 1.** Summary of barriers, facilitators, and suggested adaptations for the CST globally.

Dimension	Barriers	Facilitators	Suggested adaptations	Recurrence/influence
Language	- Multilingual communities - Non-citizens lacking local language mastery - Lack of culturally specific terminology - Ambiguous idioms/technical language	- Code-switching across local languages - Translation and back-translation - Community partners’ input on linguistic adjustments	- Use local names - Adapt concepts (e.g. joint engagement) - Simplify to everyday language - Avoid stigmatizing labels	- 10/17 studies - *Highly influential*: Community partners’ input
Persons	- Multi-child caregiving demands - Low father involvement - Limited non-specialist facilitator engagement - Immigrant acculturation challenges	- Community platforms for promotion - Local non-specialists - Multi-family member attendance - Community partners and father inclusion efforts	- Engage stakeholders and fathers - Community-based recruitment - Use cultural brokers and parent facilitators	- 12/17 studies - *Highly influential*: Community partners’ involvement
Content	- Unrealistic/overly positive vignettes - Context-irrelevant content/illustrations - Overloaded sessions - Complex skills (e.g. play) - Culturally irrelevant material	- Culturally tailored content - Community partners’ feedback for relevance	- Reflect local concepts (e.g. autism causes, play) - Add topics (e.g. behavior management) - Use local vignettes - Omit misconceptions	- 9/17 studies *- Highly influential*: Culturally relevant content
Methods	- Unpopular role-plays - Text-heavy manuals are - unsuitable for illiteracy - Video recording issues - Travel time for home visits—Tech/internet limitations - Self-directed app challenges	- Group sessions for support - Hands-on activities—Standardized toy kits - Clear caregiver booklets - Live coaching - Telehealth flexibility	- Proximity-based recruitment - Use community/school/telehealth alternatives - Booster sessions - Increased coaching - Weekly calls - Prioritize oral communication - Tech for supervision/mHealth	- 14/17 studies - *Highly influential*: Telehealth/mHealth
Context	- Low literacy/DD awareness/stigma - Travel time - Childcare shortages - Family/work conflicts - Embarrassment during home visits - Facilitator non-CST workload - Online tech issues—Immigrant barriers (acculturation, socioeconomic)	- Childcare and snacks at sessions—Online home attendance—Transportation support—Institutional backing—Community partnerships	- Provide childcare/transportation - Secure institutional support - Address acculturation/socioeconomic issues - Enhance app features - Use telehealth/hybrid formats - Reduce stigma via cultural support	- 15/17 studies - *Highly influential*: Childcare/transportation; Telehealth (14/17)

After removing 17 duplicates, 207 citations were screened by title and abstract, resulting in 30 studies for further evaluation. Forward searches using Google Scholar’s cited feature added three more citations. Following a full-text review by the first and second authors, 16 studies were excluded (3 review papers, 3 protocols, 1 proceedings, and 7 not adaptation or feasibility studies). Manual reference checks of eligible studies identified no additional citations (Figure 1).

### Participatory methods statement

This scoping review involved synthesizing and analyzing existing published literature on cultural adaptations, facilitators, and barriers in the CST program. As a secondary analysis of prior studies, it did not include primary data collection, coproduction, or direct consultation with community representatives, such as autistic individuals, caregivers, or stakeholders from the autism community.

### Positionality statement

Our research team, comprising psychologists, social workers, and doctoral students based in US institutions, brings diverse cultural backgrounds and global expertise to address autism disparities. Lead author C.M-N., a Latina from Venezuela, draws on LMIC experiences for equitable interventions like CST. Team members M.C.M. (Uruguay), A.C.R. (Mexico), V.V., and L.M.C. offer firsthand work with underserved autistic communities. P.D. contributes scaling efforts in under-resourced areas worldwide, including Africa and the Caribbean. S.D. provides adaptation expertise for CST and PMIs in Egypt and Ethiopian/Eritrean US immigrant groups, rooted in social justice. Our global perspectives—spanning Latin America, Africa, and diasporas—inform culturally responsive approaches to developmental disabilities and methodologies. We acknowledge high-resource Western positions may influence LMIC interpretations, prioritizing task-shifting and barrier reduction to amplify marginalized voices through unbiased synthesis.

## Results

### Study characteristics

Following a PRISMA-guided selection process, 17 studies met the inclusion criteria, evaluating the adaptation, feasibility, and acceptability of the CST program across diverse global contexts, with details in Supplemental Table 1. These studies were conducted in Asia (41%; *n* = 7), including Hong Kong (Lau et al., 2022; Wong et al., 2022), India (Sengupta et al., 2023), and Taiwan (Seng et al., 2022); Africa (24%; *n* = 4), specifically Ethiopia (Tekola et al., 2020; Zerihun et al., 2024), Egypt (Dababnah et al., 2025b), and South Africa (Schlebusch et al., 2024); in Europe (18%; *n* = 3) Italy (de Leonardis et al., 2025; Ferrante et al., 2024; Salomone et al., 2022; Vanoncini et al., 2025) and Serbia (Glumbic et al., 2022); and North America (18%; *n* = 3) in the United States (Dababnah et al., 2025b; Montiel-Nava et al., 2022; Ramirez et al., 2025; Zeleke et al., 2024), reflecting broad geographic diversity.

A total of 397 families participated across studies, with sample sizes ranging from 7 to 91 families. Participants were primarily recruited through community partnerships and organizations (Ramirez et al., 2025; Sengupta et al., 2023; Tekola et al., 2020; Zeleke et al., 2024). Most studies targeted children aged 2–6 years (de Leonardis et al., 2025; Lau et al., 2022; Salomone et al., 2022; Seng et al., 2022), while others adjusted to older cohorts due to delayed autism diagnoses, such as in Ethiopia, where participants were aged 4–12 years (Tekola et al., 2020; Zerihun et al., 2024), and among Ethiopian/Eritrean immigrants in the United States, where ages ranged from 2 to 9 years (Dababnah et al., 2025b; Zeleke et al., 2024). Autism was the primary diagnosis in all studies; however, over 58% (*n* = 10) also included children with other neurodevelopmental conditions, such as intellectual disabilities, communication disorders, and cerebral palsy (Dababnah et al., 2025b; Schlebusch et al., 2024; Seng et al., 2022; Sengupta et al., 2023; Tekola et al., 2020; Zerihun et al., 2024).

CST delivery included 59% (*n* = 10) in-person group sessions and home visits (e.g. Tekola et al., 2020), while 35% (*n* = 6) adopted hybrid/virtual formats to address COVID-19 challenges (e.g. Ferrante et al., 2024). Telehealth and mHealth apps (e.g. Vanoncini et al., 2025) enhanced accessibility. Study designs comprised 53% (*n* = 9) mixed-methods (e.g. Lau et al., 2022), 29% (*n* = 5) pre- and post-test (e.g. Ferrante et al., 2024), and 18% (*n* = 3) qualitative approaches (e.g. Vanoncini et al., 2025). Master trainers (MTs) and facilitators, detailed in Supplemental Table 3, included specialists,^1^ in 71% of studies, such as clinical psychologists, occupational therapists, child neuropsychiatrists, Board Certified Behavior Analysts (BCBAs), and early intervention teachers (e.g. Montiel-Nava et al., 2022; Seng et al., 2022), and non-specialists in 47%, comprising community health workers, educators, mothers of autistic children, and public health employees (e.g. Dababnah et al., 2025b; Zeleke et al., 2024; Zerihun et al., 2024). A hybrid model, combining specialists like psychiatrists with non-specialists like rehabilitation workers, was used in 53% of studies (e.g. Tekola et al., 2020). Supervision, reported in 59% of studies, featured hands-on methods like supervised practice (e.g. Ferrante et al., 2024) and TeleECHO sessions (e.g. Montiel-Nava et al., 2022), while 47% assessed fidelity with tools like the Adult-Child Interaction Fidelity Scale, achieving 62%–98% scores (e.g. Lau et al., 2022), though 53% lacked fidelity data (e.g. Tekola et al., 2020).

#### Training and non-specialist involvement

Variations in non-specialists’ qualifications and experience significantly shaped CST training and implementation across the 17 studies. Facilitators included prior experience with children or community roles, which boosted confidence and trust, as observed in Ethiopia, where local health workers leveraged cultural knowledge—drawing on local customs, traditions, and social norms—to improve attendance (Tekola et al., 2020; Zerihun et al., 2024). Higher educational attainment (e.g. college-educated facilitators in India) facilitated quicker mastery of CST strategies, shortening training periods (Sengupta et al., 2023). Barriers encompassed limited autism-specific knowledge, requiring extended training (e.g. 6–12 weeks in South Africa; Schlebusch et al., 2024) and variable fidelity (62%–98% across sites (Lau et al., 2022; Sengupta et al., 2023). Low literacy or lack of prior training in LMICs necessitated oral adaptations and additional supervision (Zerihun et al., 2024). These findings suggest that tailored training and ongoing support are critical to address qualification disparities and enhance CST effectiveness.

#### Adaptations and outcomes

CST cultural adaptations were implemented across language, content, and format domains to ensure cultural relevance and feasibility, as synthesized from the 17 studies (see Supplemental Table 3). These adaptations addressed recurring barriers and were highly influential in enhancing CST global applicability.

*Language and Terminology*: Most studies translated CST materials into local languages, including Italian (de Leonardis et al., 2025; Ferrante et al., 2024; Salomone et al., 2022; Vanoncini et al., 2025), Serbian (Glumbic et al., 2022), Chinese (Lau et al., 2022; Seng et al., 2022; Wong et al., 2022), Hindi and Marathi (Sengupta et al., 2023), Amharic (Dababnah et al., 2025b; Tekola et al., 2020; Zeleke et al., 2024; Zerihun et al., 2024), Arabic (Dababnah et al., 2025b) Spanish (Ramirez et al., 2025), and English (Montiel-Nava et al., 2022;). In multilingual contexts like South Africa, India, and Taiwan, facilitators integrated code-switching^2^—alternating between languages within conversations—to enhance comprehension (Schlebusch et al., 2024; Seng et al., 2022; Sengupta et al., 2023). The Ethiopian immigrant study in the United States (Dababnah et al., 2025b; Zeleke et al., 2024) also discussed that the parents incorporated some English into the sessions, although it was primarily delivered in Amharic. Translations were tailored to reflect culturally specific concepts, such as “joint engagement,” with notable adjustments in Hong Kong using traditional Chinese terms (Wong et al., 2022), simplified Chinese booklets in Taiwan (Seng et al., 2022), and Latino values like *personalismo* in Spanish adaptations (Ramirez et al., 2025). In Italy, Salomone et al. (2022) adjusted for linguistic gaps, while sites like India (Sengupta et al., 2023), Ethiopia (Tekola et al., 2020), and the United States (Zeleke et al., 2024) changed character names in booklets to align with local conventions. Community partners’ input, noted in 10/17 studies, was a highly influential driver of these linguistic adaptations.

*Content Simplification*: Twelve studies, particularly in low-literacy settings like Ethiopia (Tekola et al., 2020; Zerihun et al., 2024) and Egypt (Dababnah et al., 2025b) simplified materials using images, oral narratives, and culturally relevant vignettes—for example, Latino family dynamics in the work by Ramirez et al. (2025). Content was adjusted to remove irrelevant elements, such as “family sins” in Hong Kong (Wong et al., 2022), and to address local practices, like play and physical punishment in Ethiopia (Tekola et al., 2020; Zerihun et al., 2024). Unique adaptations included a caregiver well-being module in South Africa (Schlebusch et al., 2024) and toy kit distribution in Hong Kong (Lau et al., 2022). Tekola et al. (2020) also adapted home visits to avoid straining family resources by declining food or drink. This domain, noted in 9/17 studies, was highly influential due to its focus on cultural relevance and caregiver engagement.

*Delivery Format and Technology*: Fourteen studies adopted hybrid and telehealth formats to overcome logistical constraints and pandemic-related disruptions (e.g. Ferrante et al., 2024; Dababnah et al., 2025b; Montiel-Nava et al., 2022; the United States; Zeleke et al., 2024), with virtual home visits enhancing flexibility (Glumbic et al., 2022; Montiel-Nava et al., 2022). Pre-recorded videos, used in six studies (Ferrante et al., 2024; Lau et al., 2022; Montiel-Nava et al., 2022; Seng et al., 2022; Wong et al., 2022), replaced live demonstrations and included wellness activities to support caregivers (Lau et al., 2022), with enhanced feedback mechanisms improving fidelity (Ferrante et al., 2024). An innovative mHealth app with gamification and customization, developed by Vanoncini et al. (2025) and de Leonardis et al. (2025), emerged as a promising adaptation for self-directed learning.

#### Challenges, facilitators, and adaptations

CST implementation encountered multifaceted challenges across technical, cultural, logistical, instructional, and systemic domains, as detailed in Table 1. Technical barriers emerged prominently with the shift to telehealth during the COVID-19 pandemic, including increased attrition, limited Internet access, and caregiver difficulties with technology (Lau et al., 2022; Ferrante et al., 2024; Glumbic et al., 2022). Caregivers using e-learning platforms reported a lack of feedback as a key limitation, while others struggled with recording child interactions (Lau et al., 2022; Wong et al., 2022).

Cultural challenges, identified in 15/17 studies, included low autism awareness and high stigma, particularly hindering uptake in regions like Ethiopia and India (Sengupta et al., 2023; Tekola et al., 2020; Zerihun et al., 2024). Unfamiliarity with CST strategies such as praise and play clashed with local parenting practices, while privacy concerns during home visits caused discomfort for some families (Ferrante et al., 2024). Logistical barriers, also prevalent in 15/17 studies, encompassed transportation challenges, scheduling difficulties for home visits (Salomone et al., 2022; Sengupta et al., 2023; Tekola et al., 2020), and a lack of childcare, which limited participation in group sessions (Tekola et al., 2020; Wong et al., 2022). Instructional issues, noted in 14/17 studies, arose from text-heavy materials reducing accessibility in low-literacy settings (Schlebusch et al., 2024; Zerihun et al., 2024) and poorly received role-play activities or overly simplistic demonstrations (Montiel-Nava et al., 2022; Sengupta et al., 2021). Systemic challenges, affecting 8/17 studies, included facilitators’ non-CST workloads and insufficient institutional support, undermining program sustainability (Salomone et al., 2022; Seng et al., 2022).

Adaptations to home visits were a key aspect of format modifications in several studies, with sites implementing alternatives such as conducting visits in community centers, schools, or via telehealth to enhance feasibility in resource-limited or rural areas (Sengupta et al., 2023; Lau et al., 2022; Schlebusch et al., 2024). In response to the COVID-19 pandemic, geographic dispersal of participants, and logistical constraints, multiple implementations shifted individual sessions (typically home visits) to remote formats using video conferencing, phone calls, or hybrid models, which proved acceptable and feasible while maintaining program fidelity (Dababnah et al., 2025a, 2025b; Ramirez et al., 2025; Zeleke et al., 2024). However, facilitators commonly encountered challenges during home visits, including logistical issues such as extended travel times, coordination difficulties, transportation costs, and constraints in rural or low-resource settings where distances between homes are significant (Dababnah et al., 2025b; Schlebusch et al., 2024; Tekola et al., 2020; Zerihun et al., 2024). Caregiver engagement was also hindered by factors like reluctance to participate in video-recorded interactions for practice or feedback, competing family responsibilities, limited childcare options, and scheduling conflicts (Sengupta et al., 2023; Wong et al., 2022; Zeleke et al., 2024). Cultural barriers further complicated implementation, encompassing stigma around developmental disabilities that discouraged open discussions in home environments, privacy concerns in multigenerational households, low literacy affecting comprehension of materials during visits, and environmental constraints like small living spaces or distractions from other family members (Glumbic et al., 2022; Montiel-Nava et al., 2022; Ramirez et al., 2025; Tekola et al., 2020). Additional challenges in immigrant communities included limited technology skills, inconsistent Internet access, and the need to ship physical materials for participants joining remotely via phone (Dababnah et al., 2025b; Zeleke et al., 2024). These challenges underscore the need for tailored support systems, such as booster sessions, weekly phone check-ins, institutional resources for transportation, and specialized training on remote delivery to improve facilitator efficacy and program sustainability.

Despite these obstacles, several facilitators enhanced CST implementation (Table 1). The transition to online delivery, a highly influential factor in 14/17 studies, alleviated travel and childcare burdens, boosting caregiver engagement (Ferrante et al., 2024; Glumbic et al., 2022; Montiel-Nava et al., 2022). For in-person sessions, transportation support and reduced geographical catchment areas facilitated participation in Ethiopia and India (Sengupta et al., 2023; Tekola et al., 2020). Weekly follow-up calls, implemented in various contexts, supported home practice troubleshooting (Seng et al., 2022). Group sessions, valued in 10/17 studies, provided emotional support and peer connection (Glumbic et al., 2022; Salomone et al., 2022; Schlebusch et al., 2024; Zerihun et al., 2024). Community partners’ involvement and community resource utilization, recurring in 13/17 studies, were pivotal facilitators, especially in Ethiopia and South Africa (Schlebusch et al., 2024; Tekola et al., 2020).

## Discussion

Global disparities in autism intervention research persist, with studies heavily concentrated in HICs (Gillespie-Lynch & Brezis, 2018), excluding most autistic children in LMICs, where poverty and inadequate healthcare limit access (Olusanya et al., 2018; Zerihun et al., 2024). CST scalable, culturally adaptable framework empowers caregivers, addressing barriers (World Health Organization (WHO), 2022). This review identified 17 CST studies, mostly in LMICs, enhancing global research diversity.

CST flexibility enables cultural and linguistic adaptations, aligning with diverse families’ values and needs (Salomone et al., 2019). In Ethiopia, verbal instruction and visual aids replaced text-heavy materials for low-literacy populations (Tekola et al., 2020; Zerihun et al., 2024). In Indigenous communities, culturally relevant images and oral transmission, like storytelling, enhance engagement by reflecting local learning styles (Balcazar et al., 2010; Mbanda et al., 2021; McCarty, 2003). In rural Pakistan, simplified oral content and visual aids improved accessibility (Hamdani et al., 2021). Culturally adapted interventions improve adherence and outcomes (BigFoot & Funderburk, 2011; McCarty, 2003). PMIs should prioritize oral delivery and visual aids to address literacy barriers, foster engagement with diverse learning styles, promote culturally congruent practices, and empower families, enhancing developmental outcomes for children with autism.

CST’s cultural adaptations extended beyond literacy changes to address societal beliefs and caregiving norms to enhance acceptability. In Ethiopia, CST countered harmful autism misconceptions, like beliefs in cures or the use of physical punishment, and promoted positive practices (Tekola et al., 2020). In India, it incorporated strategies to reduce stigma and aligned with familial expectations (Sengupta et al., 20231). CST balances fidelity with flexibility, maintaining core content while adapting for relevance. Chinese adaptations used culturally appropriate terminology for joint engagement, while Ethiopian adaptations replaced picture-based schedules with gestures (Tekola et al., 2020; Wong et al., 2022). These adaptations ensure relevance and sustainability for diverse autism populations, supporting equitable intervention delivery while maintaining its foundational principles.

The review also identified key systemic barriers to CST implementation, including logistics, resources, and technology access. Transportation barriers are well-documented in PMIs, disproportionately affecting families from lower socioeconomic backgrounds who face long travel distances and limited public transportation options (Daniels et al., 2017; Wainer & Ingersoll, 2015). To mitigate these challenges, CST has successfully integrated telehealth delivery formats, reducing geographic constraints and enhancing accessibility (Lau et al., 2022; Ferrante et al., 2024). However, limited Internet access in LMICs challenges telehealth delivery (Sengupta et al., 2023; Tekola et al., 2020). Addressing digital divides is critical for equitable CST access, particularly as telehealth becomes an increasingly integral component of global health initiatives.

Incorporating community partnerships emerged as another critical facilitator of CST’s success. Community involvement ensures cultural relevance and fosters trust between facilitators and families, which is essential for program acceptance. For example, local partners played a central role in adapting CST materials to align with family routines and values, promoting caregiver engagement and sustainability (Sengupta et al., 2023). Task-shifting models, where non-specialist facilitators such as community health workers or teachers deliver the intervention, further enhance CST’s scalability and feasibility in resource-limited settings (Rahman et al., 2016; Schlebusch et al., 2024). By leveraging local personnel, CST addresses the shortage of trained specialists in LMICs while empowering communities to take ownership of the intervention.

However, task-shifting also presented challenges, including the risk of overburdening non-specialist facilitators and variability in training quality and supervision. Non-specialist facilitators often juggle multiple responsibilities, making it difficult to maintain intervention fidelity without adequate support systems (Javadi et al., 2017). To address this, robust training protocols and ongoing supervision are essential, as adherence to delivery standards directly influences program outcomes (Carroll et al., 2007; Durlak & DuPre, 2008). Yet, inconsistencies in fidelity monitoring and supervision across implementation sites remain a significant limitation. Only one study, conducted in Canada, has systematically examined CST training and fidelity processes, revealing high attrition rates and underscoring the need for rigorous evaluative frameworks (Ibrahim et al., 2024). Future research should prioritize understanding the factors that influence facilitator retention and program fidelity, particularly in low-resource contexts.

Nearly all existing studies report CST being delivered by specialist health professionals, with few exceptions. Scaling the program will require non-specialists to serve as facilitators, particularly in low-resource countries. Nonetheless, the majority of the included studies (e.g. Ferrante et al., 2024; Salomone et al., 2022; Settanni et al., 2024) report CST delivery by specialist health professionals, such as clinicians, therapists, or psychologists, often within public health or clinical settings in HICs like Italy or structured systems in Hong Kong. This reliance on specialists limits the scalability of CST, particularly in LMICs and underserved regions where access to trained professionals is scarce. Only a subset of studies explored non-specialist delivery, such as community workers and teachers in India (Sengupta et al., 2023), or local facilitators in Ethiopia (Tekola et al., 2020; Zerihun et al., 2024). This predominance of specialist-led interventions represents a significant limitation, as it restricts the program’s applicability in resource-constrained settings where non-specialists are critical for scalability.

Several studies provide preliminary insights into non-specialist facilitation of CST, offering a foundation for addressing scalability. This study highlighted the effectiveness of cascade training models, where MTs (specialists) train non-specialists, enabling wider reach in low-resource settings. Similarly, Sengupta et al. (2023) in India demonstrated the feasibility of using community workers and teachers as facilitators, leveraging their local knowledge to enhance cultural relevance, though challenges included discomfort with interactive methods like role-play among less experienced non-specialists. In Ethiopia, Tekola et al. (2020) and Zerihun et al. (2024) utilized local non-specialist facilitators, supported by simplified materials (e.g. Amharic translations, visual aids) and supervision, to address low literacy and stigma in rural communities. Schlebusch et al. (2024) in South Africa also trained non-specialists to deliver Acceptance and Commitment Therapy (ACT)-based CST modules, noting that simplified delivery (e.g. code-switching, illustrations) was critical for success. However, these studies also identified barriers to non-specialist delivery, including variability in qualifications, limited training duration, and resource constraints (e.g. lack of toys or technology). For instance, Tekola et al. (2020) noted that non-specialists required extended training to address cultural beliefs about disability, while Seng et al. (2022) in Taiwan reported variability in facilitator experience impacting fidelity. Fidelity measures, such as the ENACT scale (used in Hamdani et al. (2021) and Sengupta et al. (2023)) and Adult–Child Interaction Fidelity Scale (Seng et al., 2022), were critical for ensuring quality, but frequent supervision was resource-intensive, particularly for less experienced non-specialists. A limitation of this scoping review is the lack of clear evidence on the extent to which CST is currently delivered by specialists versus non-specialists across the 17 included studies. While the program is designed for task-shifting to non-specialists to address the treatment gap in LMICs, the geographic diversity of the studies (e.g. high-income settings like Italy and Hong Kong, and LMICs like India and Ethiopia) suggests variability in implementation that may hinder scalability. Further research is needed to confirm delivery patterns and their impact on accessibility.

Despite the program’s successes, methodological limitations in the 17 included studies underscore the need for enhanced rigor in exploring the cultural adaptations, feasibility, and acceptability of the CST program. While fidelity gaps (e.g. 53% of studies lacking fidelity data) and biases (e.g. language and publication restrictions) have been critiqued, the heterogeneity in study designs further introduces interpretation biases that warrant explicit consideration. For instance, the predominance of pre–post designs (29% of studies) and pilot studies, as opposed to randomized controlled trials (RCTs), may overestimate intervention effects due to the absence of control groups, potentially leading to biased conclusions about CST’s effectiveness across diverse contexts. This is particularly evident in studies with small sample sizes, such as (Dababnah et al., 2025b), which involved only 25 Eritrean and Ethiopian immigrant mothers in a single-arm pilot delivered remotely. Although the study demonstrated promising improvements in parental knowledge, self-efficacy, and child communication outcomes, the limited sample size and lack of randomization restrict generalizability to broader immigrant contexts, where factors like acculturation levels, socioeconomic variability, and access to technology could influence results. Larger-scale RCTs, incorporating diverse subgroups within immigrant populations, are essential to mitigate these biases and provide more robust evidence for CST’s scalability in underserved settings.

The restriction to English and Spanish publications introduces a language bias, potentially excluding studies in languages like Amharic or Hindi, which are critical for understanding cultural adaptations in Ethiopia and India. This may underrepresent region-specific strategies and community partners’ experiences, narrowing the review’s scope. In addition, the reliance on published literature may introduce publication bias, favoring studies with positive feasibility outcomes and overlooking gray literature that could reveal challenges in acceptability or adaptation. RCTs underway in Ethiopia and Kenya, as of 24 August 2025, focus on non-specialist delivery models and will help identify core CST components for cultural fidelity (Nortey et al., 2024). The comprehensiveness of this review may be limited by the absence of emerging or unpublished studies not captured in our database searches. For example, ongoing trials, such as a Brazilian online CST implementation and a community-based CST study in India, presented at recent conferences (e.g. INSAR 2025), indicate continued evolution in CST adaptations. These studies, unavailable in peer-reviewed form as of August 2025, highlight the need for future updates to incorporate new evidence on digital and community-driven delivery models, particularly in diverse global contexts.

Future research should focus on enhancing the evidence base for CST delivery by non-specialists, particularly in LMICs and underserved settings. Priority areas include conducting rigorous trials (e.g. RCTs or hybrid studies) to assess the effectiveness of non-specialist versus specialist-led CST, building on findings from Sengupta et al. (2023) regarding child and caregiver outcomes. Optimal training models—tailored to diverse educational backgrounds with simplified, experiential formats (e.g. Schlebusch et al., 2024; Zerihun et al., 2024—)—and scalable supervision structures (e.g. remote mentoring, as suggested by Seng et al. (2022) and Wong et al. (2022)) require investigation. Development of context-appropriate fidelity tools (e.g. adapted ENACT; Glumbic et al., 2022) and studies on cultural adaptations to address stigma (e.g. (Dababnah et al., 2025b; Tekola et al., 2020) by community-based non-specialists (e.g. Zeleke et al., 2024) are also critical to ensure quality and equity.

## Conclusion

CST’s scalability as an autism intervention stems from balancing standardization with cultural adaptability, leveraging implementation science principles like task-shifting and sustainability (Salomone et al., 2019). Building community partnerships is critical to inform culturally responsive adaptations, ensuring feasibility and acceptability in diverse settings (Sengupta et al., 2023). Context-specific adaptations, such as oral delivery for low-literacy populations, enhance accessibility in LMICs (Tekola et al., 2020). Ongoing training and fidelity monitoring for non-specialists address barriers like stigma and logistics, empowering caregivers and improving child outcomes. This review underscores CST’s value for autistic individuals by identifying facilitators (e.g. telehealth, partnerships) and solutions (e.g. tailored materials) that support communication and social engagement. By fostering community-driven adaptations and bridging disparities, CST promotes equitable, inclusive autism care worldwide.

## Notes

1. For the CST, specialists refer to professionals with specialized training and in-depth knowledge of early childhood development and neurodevelopmental disorders. In contrast, non-specialists may include professionals and nonprofessionals with previous experience working with children with developmental disorders without necessarily having any formal training in the field (WHO, 2022).

2. Code-switching is a linguistic phenomenon in which a bilingual or multilingual speaker alternates between two or more languages within a single conversation, utterance, or even sentence (Mabule, 2015).
